# Everybody's Page

**Published:** 1892-05-07

**Authors:** 


### Everybody's Page.

Now that vegetables are scarce, householders should not despise the common stinging nettle as a source of food. The young shoots when cooked taste rather like very delicate turnip tops. The act of boiling, of course, takes from the nettles their power of stinging.

Those doctors who have settled so completely to their own satisfaction that the ownership of prescriptions rests with them will perhaps explain for what consideration the patient pays his fee if not for the ownership of the prescription. The doctor who prescribes for a patient and receives a fee is in a similar position to a watchmaker who makes a watch and sells it to a purchaser; he parts with his prescription for consideration received.

There is no doubt that faith works wonders, and is often more potent than drugs, and that faith without works is not altogether a desirable moral state. It is reported of an American gentleman that he awoke during the night with a feeling of suffocation, as the result of swallowing a set of false teeth. Every effort which was made to extract the offending molars proved inefficacious, and the family were summoned to the bedside of the dying man to bid their sad farewell. Whilst this ceremony was going on the teeth were found in a drawer, the farewells were postponed *sine die*, and the dying man was restored to life.

The hunting of the bacillus, which no doubt forms one of the most exciting pastimes of the scientist of the nineteenth century, and has not the drawback incidental to fox-hunting that the quarry requires preserving, has led, so it is asserted, to the discovery of the germ which is the cause of measles, and to what end? We have discovered a good many germs during the past few years without much benefit, for we seem as far as ever from knowing how to counteract their effects. Modern science does not appear to know much more about microbes than can be gleaned from Sanskrit treatises on medicine.

In the matter of women's rights Abyssinia is far ahead of benighted Europe. The wife in that country is indeed, and not in letter only, the better half. According to Signor F. Martini, the house and all its contents belong to her, and if the husband offends her she not only can but does turn him out of doors till he is duly repentant, and makes amends by the gift of a cow or the half of a camel, that is to say, we suppose, half the value of a camel. On the other hand, it is the privilege and duty of the wife to abuse her husband, and she can divorce herself from him at pleasure, whereas the husband must show reasons to justify such an act on his part. This is certainly an astounding reversal of the relationship between husband and wife, which no doubt has many recommendations in the eyes of the fair sex.

